# Accurate Device-Free Tracking Using Inexpensive RFIDs

**DOI:** 10.3390/s18092816

**Published:** 2018-08-27

**Authors:** Liyao Li, Chongzheng Guo, Yang Liu, Lichao Zhang, Xiaofei Qi, Yuhui Ren, Baoying Liu, Feng Chen

**Affiliations:** School of Information Science and Technology, Northwest University, Xi’an 710127, China; liliyao@stumail.nwu.edu.cn (L.L.); guo_chongzheng@stumail.nwu.edu.cn (C.G.); nwu_ly729@stumail.nwu.edu.cn (Y.L.); zhanglichao0331@gmail.com (L.Z.); qixf@nwu.edu.cn (X.Q.); ryhui@nwu.edu.cn (Y.R.)

**Keywords:** RFID, device-free tracking system, Doppler frequency shift, path matching model

## Abstract

Without requiring targets to carry any device, device-free-based tracking is playing an important role in many emerging applications such as smart homes, fitness tracking, intruder detection, etc. While promising, current device-free tracking systems based on inexpensive commercial devices perform well in the training environment, but poorly in other environments because of different multipath reflections. This paper introduces RDTrack, a system that leverages changes in Doppler shifts, which are not sensitive to multipath, to accurately track the target. Moreover, RDTrack identifies particular patterns for fine-grained motions such as turning, walking straightly, etc., which can achieve accurate tracking. For the purpose of achieving a fine-grained device-free tracking system, this paper builds a trajectory estimating model using HMM (Hidden Markov Model) to improve the matching accuracy and reduce the time complexity. We address several challenges including estimating the tag influenced time period, identifying moving path and reducing false positives due to multipath. We implement RDTrack with inexpensive commercial off-the-shelf RFID (Radio Frequency IDentification) hardware and extensively evaluate RDTrack in a lobby, staircase and library. Our results show that RDTrack is effective in tracking the moving target, with a low tracking error of 32 cm. This accuracy is robust for different environments, highlighting RDTrack’s ability to enable future essential device-free moving-based interaction with RFID devices.

## 1. Introduction

Have you ever heard news that jewelry stores or museums have been robbed? Has your house been visited by a thief? It is common for some victims to go to a police station to report crimes with respect to their property and security. Tracking [1] intruders and protecting private property from the illegal encroachment of intruders is a major problem in museums, jewelry stores and warehouses where the items contained therein are very valuable. In real life, we envision target tracking as an essential means to support anti-intrusion protection. Existing approaches based on vision [2], ultra-sound [3] and infrared [4] have been applied in target tracking. While promising, these techniques have various limitations such as requiring line-of-sight, sensitivity to lighting, short communication distance, etc.

With the ubiquity of RF-enabled devices and infrastructure, some RF-based device-free target tracking systems [5,6,7] have been proposed recently to overcome the above limitations in order to support anti-intrusion protection. Device-free tracking systems hosted on inexpensive devices leverage the Received Signal Strength Indicator (RSSI) [5,8] or Channel State Information (CSI) [7,8,9,10] as fingerprints, which are easily corrupted by environmental changes. However, the equipment based on RSSI, e.g., sensor nodes, is easily influenced by multipath. In addition, the equipment based on CSI, e.g., WiFi, not only need fingerprints and have a high-cost of deployment, they also are easily influenced by multipath. What is more, the equipment is based on Frequency-Modulated Continuous Wave (FMCW) [6], e.g., Universal Software Radio Peripheral (USRP) and Wireless Open Access Research Platform (WARP), while the high cost is a major issue hindering its wide deployment.

Thanks to the rapid development of the Internet of Things (IoT), the RFID technique is widely used in many areas, like Wal-Marts and museums. RFID-based target tracking systems [11,12] are based on analyzing the changes in the characteristics of wireless signals caused by human motion, such as the phase or Doppler shift. However, due to the complex wireless propagation and the noisy wireless channel, these systems require mass-deployed tags and ceaseless calibration. Moreover, none of these systems provide fine-grained movement of the target.

**Figure 1:**
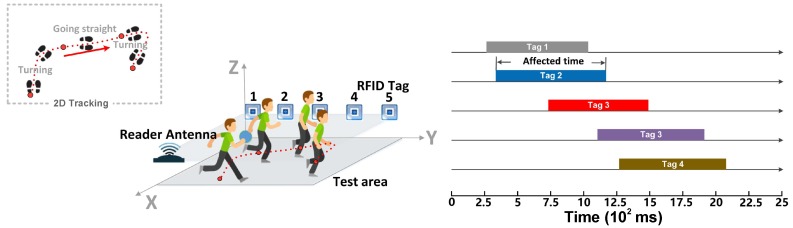
The function of RDTrack.

This paper introduces RDTrack, a fine-grained RFID tracking system that can work in the presence of rich multipath. RDTrack can adapt to different environments without any training and is able to operate in non-line-of-sight scenarios. The basic idea is to utilize the frequency variation of the motion on the backscattered RF signal received by the RFID reader from the RFID tag to identify the trajectory of the target, as is shown in Figure 1.

Unlike previous device-free target tracking systems, which leverage the change in multipath or ignore the change in multipath to detect trajectories and cannot adapt to multiple environments, RDTrack breaks the routine and estimates accurate Doppler shift, which is insensitive to multipath.

**Figure 2:**
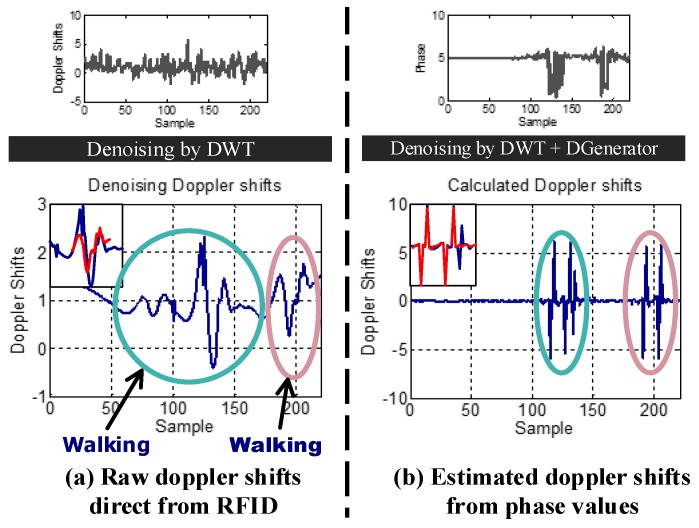
The Doppler shifts we calculated were better than we obtained from the RFID reader (DGenerator).

How do we obtain accurate Doppler shifts from RFID systems? It is quite difficult to estimate Doppler shifts from inexpensive devices. Although the raw Doppler shifts can be obtained from the RFID system directly, they are too noisy to track human motion due to hardware imperfections. As shown in Figure 2, the raw Doppler shifts have random noise. The Doppler values after denoising from one tag are quite different when the target walks straight, twice on the same path. To address this problem, RDTrack extracts accurate phase values from the raw signal and leverages phase changes to calculate the Doppler shifts. Based on the Discrete Wavelet Transform (DWT) [13,14,15], we use the heursure [16,17,18] threshold method to remove the noisy part from the raw phase values. Figure 2 demonstrates that Doppler shifts that we calculate from the phase values are more accurate to measure human motion.

We take advantage of the estimated Doppler shifts and build Doppler profiles to describe different motions of the target (such as walking, turning, etc.). Another challenge is how to determine the beginning and ending of each motion. To address this challenge, RDTrack leverages the phenomenon [11] that the frequency has a significant change when a human moves around the line-of-sight between the reader antenna and the tag. To be specific, RDTrack obtains the influenced time of each tag by detecting the points of mutation from the first detail coefficients, which are divided from the raw signal by using the DWT technique. After that, RDTrack can infer the main trajectory from the sequence of affected tags.

Finally, how can we get the fine-grained trajectory? To address this problem, RDTrack leverages data normalization techniques that can make the denoised data in the same dimension, which increases the cohesion of entity types. In addition, RDTrack leverages boosted decision tree regression, which turns discrete data into a regression curve, in order to obtain its speed and response time, as well as improve its scalability and modularity. Furthermore, we introduce a trajectory estimating model based on HMM to identify the fine-grained PIU (Path Information Unit; PIU means each motion segment of the target), which helps improve tracking accuracy and robustness.In the end, matching the specific PIU with the profiles helps us to get the fine-grained trajectory, which has a linear time complexity.

We implement RDTrack with an ImpinjR420 reader, which supports four 9 dbi-gain directional antennas (9028PCL12NF). We use the Impinj H47 passive tags in our experiments. The frequency of the reader is 924.375 MHz, and the bandwidth is 231.25 kHz. Meanwhile, four realistic scenarios are tested covering more than 1000 paths. Our results reveal that RDTrack can track the moving target with a low tracking error of 32 cm. Amazingly, the tracking error will decrease to 27 cm if we use two RFID systems. RDTracks can work in other different environments, highlighting its ability to enable future essential device-free motion-based interaction with RFID devices.

Contributions: This paper makes the following contributions:

- It presents a device-free tracking scheme that exploits Doppler shifts as a feature of the target’s motion. As a result, the design delivers fine-grained resolution in different environments with different multipath conditions.
- It is the first to demonstrate the capability of the DWT technique to detect the beginning and the ending of the influenced time for each tag caused by the target motion, and we successfully use it to track the target. While our scheme has been verified in the context of RFIDs, the basic idea can be extended to other tracking systems.
- It presents a less complex trajectory estimation model, which has a high tracking accuracy of 32 cm.
- It demonstrates a prototype system and evaluates it in real-world deployments.

## 2. Background and Analysis

Ultra-High Frequency (UHF) RFID systems communicate using backscatter. The RFID reader transmits a high power RF signal, and nearby RFID tags reply to the RFID reader’s query by reflecting the high power RF signal using ‘On’ and ‘Off’ states. The tags transmit a ‘1’ bit to change their antennas’ impedance to reflect the reader’s RF signal. In contrast, the tags transmit a ‘0’ bit to remain in their initial silent state [19]. With the backscattered signal reflected from tags, the RFID reader can acquire some information about tags, including Tag ID, RSS [20], phase and Doppler shifts.

### 2.1. Backscatter Communication

A typical passive RFID system is made up of the following components: the RFID reader with multiple antennas or interrogators, the passive RFID tag and the back-end monitor platform. Passive tags do not have any power supply. Through the tag antenna, the magnetic or electromagnetic field of the RFID reader provides all the energy required for operating the tag [19]. This means that the energy emitted by the RFID reader is used for data transmission both from the reader to the tag and back to the RFID reader. If the tag is located outside the RFID reader’s communication range, the tag has no power supply at all and will not be able to send signals. Two points about passive RFID system are particularly relevant to the tracking problem.

- There is no carrier frequency offset between the reader and the tag, because the tag does not generate its own RF signal, but rather reflects the reader’s signal [19]. Hence, the reader can use coherent detection to recover the complex channel values of the tag. These channels are available to the tracking systems and are used by many proposals [11,12,21,22], as well as ours.
- The passive RFID system’s range is limited by the reader’s sensitivity to the reflected signal and by the amount of energy that reaches the tag from the reader. The actual return signal is tiny as a result of two attenuations, including the first attenuation occurring as EM waves [19] radiating from the reader to the tag and the second attenuation occurring when reflected waves come back from the tag to the reader. Therefore, the returning energy is $\frac{1}{r^{4}}$, where *r* is the distance between the tag and the reader. Providing accurate tracking information while keeping the range large will be the valuable and necessary features of RFID systems.

### 2.2. Signal Characteristics

**Figure 3:**
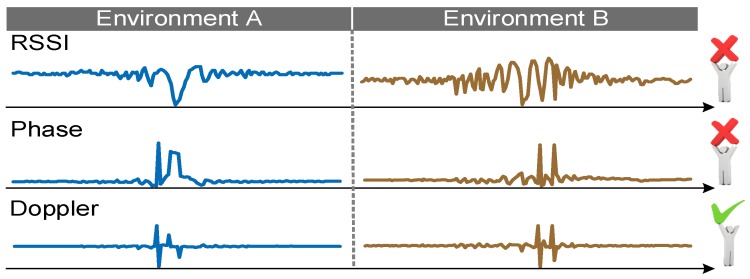
RSSI, phase and Doppler analysis in different environments.

As mentioned before, we can get RSS (Received Signal Strength), phase and the Doppler frequency shift according to backscattered information about tags. As shown in Figure 3, RSS and phase are sensitive to environment changes, whereas Doppler is not sensitive to environment changes. In addition, as shown in Figure 2, compared with the Doppler shifts extracted from the reader directly, the Doppler shifts that we calculated from the phase changes have absolute advantages.

Due to the presence of human motions, the Doppler shifts can be caused by the phase change data during a diminutive sampling interval, and each PIU has its own variation. Hence, we mainly use the phase (RF phase) and Doppler (Doppler frequency shift).

#### 2.2.1. RF Phase

The RF phase is a periodic function of wavelength (λ) and distance (*d*) with period 2π. The phase (θ) can be calculated by:

(1) $$\left\{\begin{array}{c} \theta =(\frac{2\pi }{\lambda }\cdot 2d+\theta_{noise})\;\mathrm{mod}\;2\pi \\ \theta_{noise}=\theta_{TX}+\theta_{RX}+\theta_{tag} \end{array}\right.$$

where $\theta_{noise}$ is the system noise, which represents the phase rotation induced by $\theta_{TX}$ as the reader’s transmitter circuit, $\theta_{RX}$ as the reader’s receiver circuit and $\theta_{tag}$ as the tag’s reflection characteristic, respectively. The reader can utilize the phase value to calculate the offset from the received signal and the sent signal.

#### 2.2.2. Doppler Frequency Shift

Doppler frequency shift is the shift of the frequency from the backscattered signal due to relative movements between the reader and the tag. If Δt represents the time duration and Δθ represents the phase offset, the Doppler frequency shift ($D_{shift}$) can be derived by:

(2) $$D_{shift}=\frac{\Delta \theta }{4\pi \Delta t}$$

As mentioned before, Doppler frequency shift is closely related to the movement between the reader and the tag. Indeed, it is related to the velocity of the movement and the direction of the motion. The Doppler frequency shift $D_{shift}$ can be expressed as:

(3) $$D_{shift}=\frac{2V\cos(\Theta_{angle})}{\lambda }$$

where $\Theta_{angle}$ is the angle between the signal direction and the direction of the moving object and *V* is the velocity of the movement.

Obviously, if we got $D_{shift}$ before, we can obtain the corresponding relation between velocity and moving direction.

## 3. System Overview

**Figure 4:**
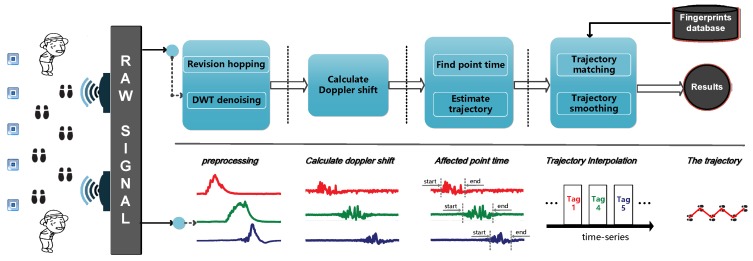
The system of RDTrack.

In this section, we introduce the macroscopical overview of RDTrack, which covers two main parts: data extraction and trajectory mapping, as illustrated in Figure 4.

Data extraction: This part concentrates on extracting the clean phase values from the raw signal, which are too noisy to use. We modify the phase values by using a self-adaption dynamic protocol method. After modifying the phase values, we denoise the phase values by using DWT.

Trajectory mapping: This part concentrates on mapping the fine-grained target movement trajectory. This is performed through three steps: segmentation, trajectory estimate and trajectory smoothing. At first, segmentation helps us excavate the period when tags are affected by the target. In addition, the trajectory estimate part helps us map the preliminary object movement trajectory referring to the tag affected time. Finally, once the beginning and the ending of the tag affected time are determined, we employ the HMM model to estimate the trajectory, and then, we can get the fine-grained trajectory.

## 4. Data Extraction

In this section, our goal is to extract the basic phase value from the raw signal and utilize the phase values to calculate the Doppler values. Because of phase ambiguity, we correct phase values firstly. Then, we employ the Discrete Wavelet Transform (DWT) [13,14,15] to reduce the noise.

After obtaining the clean phase value, we calculate the Doppler value using phase values so that we can describe different submodules in detail.

### 4.1. Revision on the Random Hopping

While the phase values we measured by the RFID reader are ambiguous, random hopping occurs when the phase values mix real phase values and ambiguous phase values.

To do this, we design a self-adaption dynamic protocol method to settle this problem of random hopping of phase values.

First and foremost, due to the phase values being in different ranges, we divide the all the phase values into different groups expressed by *P*. Then, we calculate the quantity of every group expressed by *W* and select the group($I_{k}$) that has the largest quantity.

(4) $$P=(I_{1},I_{2},\ldots ,I_{N})$$

(5) $$W=(W_{1},W_{2},\ldots ,W_{N})$$

Next, we use the group ($I_{k}$) that we select as a baseline to modify the other groups as Equation (6):

(6) $$I_{n(m)}=I_{n(m)}\pm \pi ,1\leq m\leq W_{n},1\leq n\leq N,n\neq k$$

**Figure 5:**
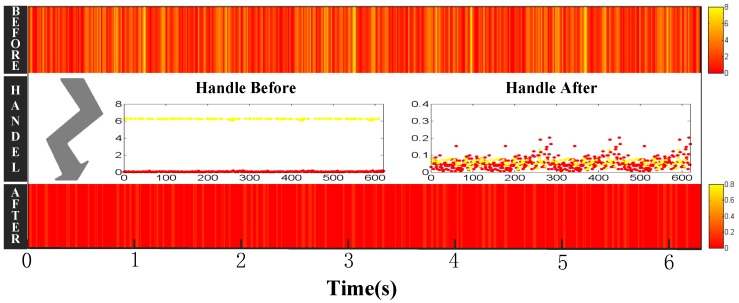
Revision on the random hopping.

Finally, we obtain the revised phase values. The modification phase values are shown in Figure 5, and the results display that our method is practical and effective.

### 4.2. Noise Reduction Using DWT

**Figure 6:**
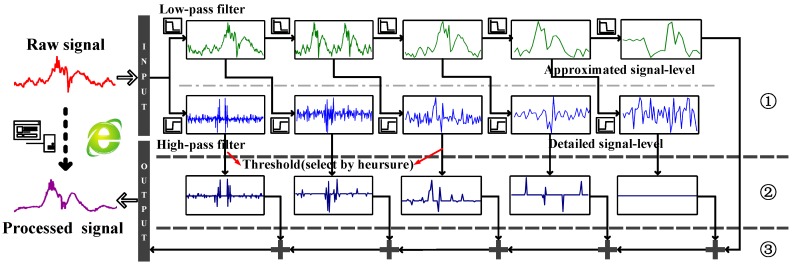
Noise reduction using DWT (① decomposition; ② threshold denoising; ③ reconstruction).

The RF signal is disturbed by the surrounding objects, and phase values after the revision step are still noisy, which affects the accuracy and robustness of RDTrack. In this part, we mainly deal with the important problem of how to get clean phase values that help us to enhance the signal-to-noise ratio and avoid false trajectories generated by other actions or noise from nearby persons in the environment. RDTrack solves this problem by using DWT, which can provide the frequency of the signal at every time. RDTrack achieves fine-grained multiscale analysis and supplies an optimal resolution in time-frequency analysis. As shown in Figure 6, wavelet-based denoising consists of three parts: decomposition, threshold denoising and reconstruction. Then, we give the details of the noise reduction submodule.

#### 4.2.1. Decomposition

Wavelet transform [13,14,15,17,23] shows a time-frequency expression of a signal. As for Discrete Wavelet Transform (DWT), it generally splits the signal into two parts: the approximation coefficients (from the low-pass filter) and the detail coefficients (from the high-pass filter) [23]. This segmentation process lasts *N* levels until the approximation coefficient cannot be decomposed. At the end, DWT splits the signal into one approximation coefficient $\alpha^{\left(N\right)}$, together with a sequence of *N* detail coefficients $\beta^{\left(1\right)},\beta^{\left(2\right)},\ldots ,\beta^{\left(N\right)}$, as shown in Figure 6. In this part, this process lasts five times. DWT coefficients at each level can be described using the following formulas:

(7) $$\alpha_{k}^{\left(N\right)}={\langle x_{i},g_{i-2^{N}k}^{\left(N\right)}\rangle }_{i}=\sum_{i\in Z}x_{i}g_{i-2^{N}k}^{\left(N\right)},N=5$$

(8) $$\beta_{k}^{\left(\zeta \right)}={\langle x_{i},h_{i-2^{\zeta }k}^{\left(\zeta \right)}\rangle }_{i}=\sum_{i\in Z}x_{i}h_{i-2^{\zeta }k}^{\left(\zeta \right)},\zeta \in \left\{1,2,\ldots ,5\right\}$$

where $\langle x_{i},h_{i-2^{\zeta }k}^{\left(\zeta \right)}\rangle$ and $\langle x_{i},g_{i-2^{N}k}^{\left(N\right)}\rangle$ are the dot product operation of two vectors, $x_{i}$ is the *i*-th input point and *g* and *h* are the Haar wavelet basis. Obviously, we use the Haar basis functions in our method.

#### 4.2.2. Threshold Denoising

The threshold denoising algorithm is effective for denoising the detail coefficients. Under the assumption of Gaussian noise, the threshold (η) is selected dynamically, and the heursure [16] threshold can minimize Stein’s unbiased risk estimator [16,17,18]. The heursure threshold integrates two threshold methods including the sqtwolog threshold [18] and the rigrsure threshold [23]. The sqtwolog threshold is a simple method that uses a fixed threshold to deal with the detail coefficients at the first two levels of which the signal-to-noise ratio is low. The fixed threshold can be calculated from:

(9) $$\eta =\varepsilon \sqrt{2logN}$$

where *N* is the length of the signal and ε is given as:

(10) $$\varepsilon =\frac{median(a)}{0.6745}\;$$

where *a* is the median value from the detail coefficient of the Doppler signal. The rigrsure threshold is the method to deal with the detail coefficients at the last three levels of which the signal-to-noise ratio is high. This method uses different SURE thresholds for different resolution levels. The SURE threshold is expressed as follows:

(11) $$\gamma_{j}=arg\min_{0<\gamma <\gamma_{u}}sure\left(\gamma ,\alpha_{j}\right)$$

where $\alpha_{j}$ is the *j*-th detail coefficient and $\gamma_{j}$ is the rigrsure threshold for $\alpha_{j}$. The function of SURE can be expressed as follows:

(12) $$sure\left(\gamma ,M\right)=i-2\cdot \theta \{j:|M_{j}\leq \gamma |\}+\left[\min(\right|M_{j}{\left|,\gamma ]\right)}^{2}]$$

where the ·θ is a operator to get the cardinality of the set $\left\{j:|M_{j}\leq \gamma |\right\}$.

#### 4.2.3. Reconstruction

Finally, after denoising the detail coefficients, we combine the approximation coefficient in the last level with all detail coefficients after denoising in this part. The reconstruction process can be described by:

(13) $$x_{i}=\sum_{k\in Z}\alpha_{k}^{\left(N\right)}g_{i-2^{N}k}^{\left(N\right)}+\sum_{\zeta =1}^{N}\sum_{k\in Z}\beta_{k}^{\left(\zeta \right)}h_{i-2^{\zeta }k}^{\left(\zeta \right)}$$

where *N* is equal to five and ζ belongs to set {1,2,…,5}.

### 4.3. Calculate Doppler Frequency Shift Using Phase Profile

Compared with using the Doppler shifts received by the reader, the method using the Doppler shifts calculated by the phase profile can remove the influence of static multipath, and it can also get cleaner Doppler shifts values. As shown in Figure 2, we can easily know that the matching degree of the Doppler shifts calculated by the phase profile is higher. Once we extract the clean phase values, we utilize the phase offset (Δθ) and time duration (Δt) to get the Doppler values ($D_{shift}$) with Equation (2).

## 5. Trajectory Mapping

In this section, our goal is to map the object movement trajectory. This is performed through three steps: segmentation, trajectory estimate and trajectory smoothing. At first, segmentation helps us excavate the period when tags are affected by the target. In addition, the trajectory estimate part helps us map the preliminary object movement trajectory referring to the tag affected time. Finally, once the beginning and the ending of the tag affected time are determined, we compare the extracted signal pattern with path profiles to find the best match using Dynamic Time Warping (DTW) [22] and then connect our trajectory to the matched pattern, so that we can get the final fine-grained trajectory. Then, we describe different submodules in detail.

### 5.1. Motion Detection and Segmentation

In this part, we mainly deal with the important problem of how to know the beginning and the ending of the tag affected time, which helps avoid false motion detection generated by other actions or noise from nearby obstacles. When somebody walks through the tag, the signal frequency from the tag will vary obviously. Utilizing the method of Section 4.2, RDTrack decomposes the signal with the db5 wavelet basis [24]. As is shown in Figure 6, DWT splits the raw signal after revising the random hopping into one approximation coefficient, together with a sequence of *N* detail coefficients.

**Figure 7:**
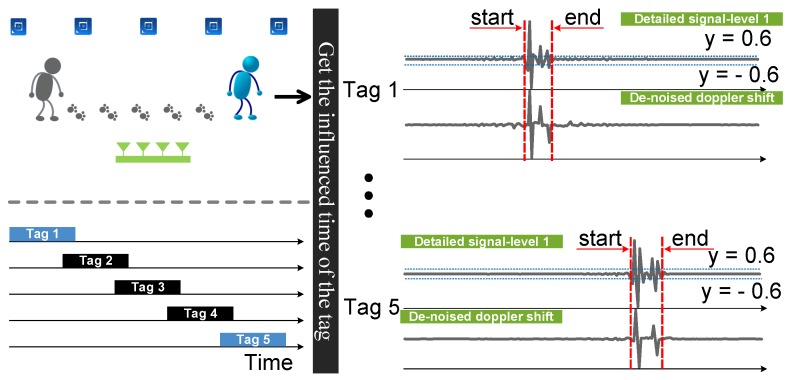
Segment the beginning and the ending of affected time of the tag.

Figure 6 shows that the signal have gaps, which can be found in detail coefficients at the first two levels. RDTrack can segment the beginning and the ending of the tag affected time by detecting gaps of the detail coefficient at the first level. As shown in Figure 7, when the detail coefficient at the first level reaches 0.6 the first time, the RDTrack considers the time to be the beginning of the tag affected time. On the contrary, when the detail coefficient at the first level reaches 0.6 the last time, the RDTrack considers the time to be the ending of the tag affected time. Obviously, we can determine that calculating the gap position with DWT is accurate.

The segmentation with DWT is universal and robust to signal features, and it is also accurate enough to show the beginning and ending of the affected time of the tag. In addition, compared with the Fourier transform, DWT is linear in time complexity.

### 5.2. Trajectory Estimate

The core of this section is to map the preliminary trajectory. RDTrack utilizes the affected time of every tag to judge the time series of affected tags, so that we can map preliminary trajectory easily. RollCaller [11] shows a phenomenon that the Doppler shiftof signals backscattered from the tag to the reader antenna has a significant change when an obstacle moves across the line of sight between the reader antenna and the tag. As shown in Figure 7, if somebody comes near the tag, the Doppler shiftof signals backscattered from the tag will be affected, and we can find the beginning and the ending affected time by detecting the mutational site. Thus, we can map preliminary object movement trajectory according to the tag affected time.

**Figure 8:**
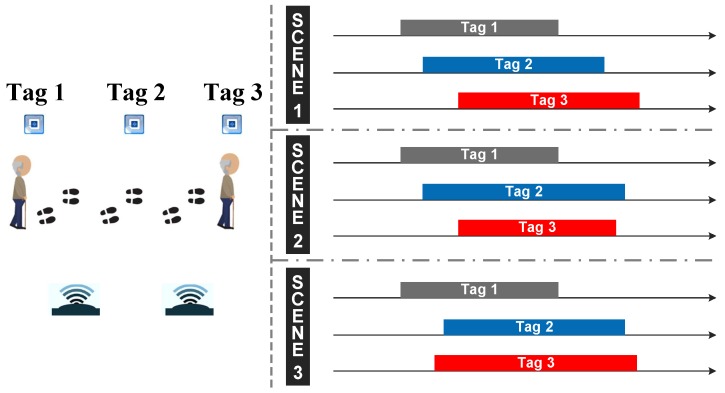
Three special targets’ affected sequence scene.

However, there are some special circumstances in the actual experiment. As is shown in Figure 8, when the person passes through the area where the tag sequence is Tag 1, Tag 2, Tag 3, the time series of three tags may overlap or coverage. In order to avoid these problems, we need to verify the time series of affected tags according to the tag affected time and the map profile, which include the location information of tags and reader antennas. This method has three advantages: (a) high-accuracy; (b) high robustness; (c) strong universality.

In some cases, due to the complex wireless propagation environment, flipping between rising and falling edges may occur. If we get the time series of affected tags only using the signal itself, the confidence level of the preliminary trajectory will be very low. RDTrack leverages the frequency to compensate for this problem. Specifically, RDTrack determines whether or not the change of adjacent data is greater than 0.6, and we get the affected time of tags by utilizing the detail approximation coefficient at the first level. The preliminary trajectory helps the system adapt to different users and different environments and, most importantly, helps the system improve the tracking accuracy.

### 5.3. Trajectory Smoothing

The most important question of RDTrack is how to get the fine-grained path information unit. Once the affected time of the tag is determined, we compare the Doppler shift values with PIU templates to find the best matching, and we add the PIU to the preliminary trajectory, so that we can get the fine-grained trajectory. In our solution, firstly, RDTrack transforms the Doppler shift values extracted from Section 5.1 into the same dimension by using data normalization, which increases the cohesion of entity types. In addition, RDTrack leverages the decision tree regression model based on AdaBoost, which turns discrete data into a regression curve. This solution can help with its speed and response time, as well as its scalability and modularity. Furthermore, we introduce a trajectory estimating model to identify the fine-grained PIU, which helps improve tracking accuracy and robustness.

#### 5.3.1. Data Normalization

In different experimental environments, the Doppler shift values may not be in the same dimension. The purpose of this section is to normalize the data from the different sources by Z-score standardization [25,26] so that the Doppler shift values extracted from Section 5.1 are in the same dimension as much as possible. Then, we normalize the primary data $f_{m}$ by using the following equation:

(14) $$f_{m\_nml}=\frac{f_{m}-\mu }{\sigma }$$

where $f_{m\_nml}$ is the normalized data, μ is the average of primary data and σ is the variance of primary data. Getting the normalized Doppler shift data prepares for establishing the HMM path matching model.

#### 5.3.2. Decision Tree Regression Based on AdaBoost

**Figure 9:**
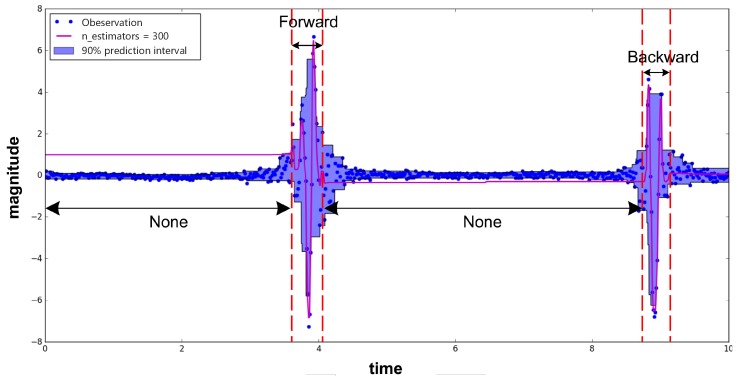
Boosted decision tree regression example.

The data after normalization, however, are still messy. We use decision tree regression [27] to smooth the data, in order to reduce the difficulty of matching. The method has obvious advantages: (a) we can model the complex and non-linear data; (b) the method does not require standardization of features and uniform quantification; and (c) the method is interpretable for the forecast results. As shown in Figure 9, RDTrack utilizes the AdaBoost algorithm [28] to improve the accuracy of decision tree regression.

#### 5.3.3. Trajectory Estimating Model Based on HMM

RDTrack presents the trajectory estimating model, RDTrack−HMM, which is based on HMM. If we split the trajectory into some parts that have different shapes of PIU including going straight, going backwards and turning, we could regard the trajectory as a PIU sequence. From this point of view, we can build the trajectory estimating model using HMM. In our trajectory estimating model, there are two states and three probability matrices. However, if the hidden state is PIU and the observable state is the wireless signal, there will be a set of observable states. Based on the above, we build the model of RDTrack−HMM as follows:

(15) λ=(S,O,π,A,B)

Therefore, we need to identify five basic parameters.

Hidden state and observable state: The hidden state *S* is the hidden state of the Markov model, where there are three states including turning, going straight and going backward. The observable state *O* is the warp path distance sequence calculated by the observable state acquisition algorithm utilizing the regression curve obtained from Section 5.3.2. Next, we introduce the observable state acquisition algorithm. $T=\{t_{1},t_{2},\ldots ,t_{\tau }\}$ is the Doppler shifts time sequence after normalization and regression analysis during tag affected time. $U=\{u_{1},u_{2},\ldots ,u_{\eta }\}$ is the corresponding Doppler shift sequence of PIU in the database.

If *T* and *U* have the same length (τ=η), we use the Euclidean distance to calculate the warp path distance:

(16) $$D_{sta}\left(t_{i},u_{i}\right)={\left(t_{i}-u_{i}\right)}^{2}$$

If (τ≠η), we build a = τ×η matrix gridding and find a warping path *W*, which is between $t_{\tau }$ and $u_{\eta }$. Then, we use the following equation to choose a least cost path $D_{W}\left(T,U\right)$:

(17) $$W=w_{1},w_{2}\ldots w_{k},max\left(\tau ,\eta \right)\leq k\leq m+n+1$$

(18) $$D_{W}\left(T,U\right)=min\left\{\sqrt{\frac{\sum_{n=1}^{k}w_{n}}{k}}\right\}$$

Finally, we can get the minimum warp path distance $D_{sta}\left(t_{i},u_{j}\right)$,and we get the most proximate corresponding PIU.

(19) $$\begin{array}{c} D_{sta}\left(t_{i},u_{j}\right)=\left.d\left(t_{i},u_{j}\right)+min\{D_{sta}\left(t_{i-1},u_{j-1}\right),\right.\\ \left.D_{sta}\left(t_{i-1},u_{j}\right),D_{sta}\left(t_{i},u_{j-1}\right)\}\right. \end{array}$$

This method of obtaining the observable state can translate the infinite signal waveform into the finite signal prototype, which reduces the space complexity and the time complexity, as well as increases the accuracy of the system.

Probability matrix: In our experiment, the initial state probability matrix is $\pi =\{\pi_{1},\pi_{2},\pi_{3}\}$, and every initial probability of hidden state is expressed as follows:

(20) $$\pi_{i}=P\left(S_{i}\right)\left(1\leq i\leq N\right)$$

In addition, the state-transition matrix is expressed by:

(21) $$A_{mn}=P\left(S_{n}|S_{m}\right)\left(1\leq m,n\leq 3\right)$$

which determines the transition of state $S_{m}$ at time t=m to state $S_{n}$ at time t=n, with the purpose of calculating the corresponding hidden states based on a given observable state. Moreover, we assume that B(S,O) is the probability of observing the symbol *O* in state *S*:

(22) $$B\left(S,O\right)=N\times \alpha_{match}+\left(D_{sta}^{max}-D_{sta}\left(S,O\right)\right)$$

where $\alpha_{match}$ is a weighting parameter, $D_{sta}^{max}$ is the maximum warp path distance, $D_{sta}\left(S,O\right)$ is the minimum warp path distance and *N* is the number of matching tags’ ID. Finally, we get the PIU at this time.

#### 5.3.4. Trajectory Detailing and Smoothing

**Figure 10:**
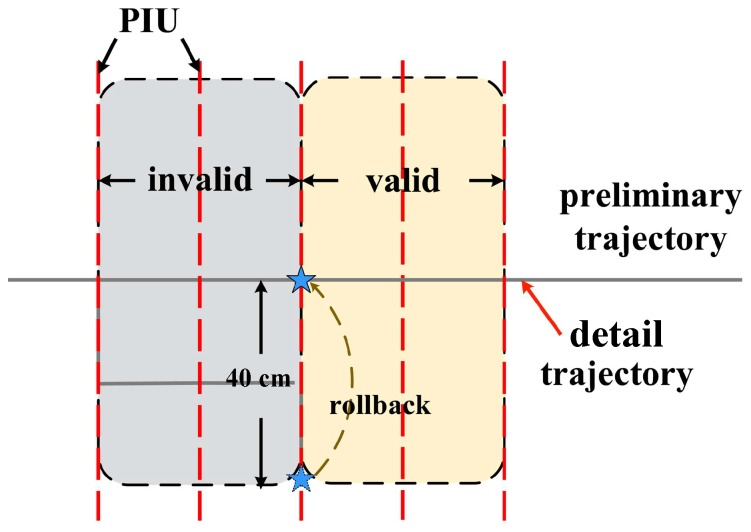
Trajectory detailing example. PIU, Path Information Unit.

Once the preliminary trajectory is determined and the PIU list is extracted, we add the PIU into the preliminary trajectory, as shown in Figure 10. RDTrack checks the PIU list periodically, adds the current PIU from the PIU list to the detail trajectory and updates the terminal point of the detail trajectory. If the terminal point of the detail trajectory diverges from the preliminary trajectory over 40 cm, RDTrack takes the terminal point of the detail trajectory rollback to the preliminary trajectory. The trajectory after adding the current PIU is not smooth, so we design an algorithm based on locally-weighted linear regression to smooth the detail trajectory. Finally, we can get the detail trajectory accurately. This method is similar to the process of resetting the tail pointer address of the linked list, which leads to space-time validity.

## 6. Implementation and Evaluation

In this section, we describe a prototype implementation of RDTrack and a performance evaluation in different experiments. We conduct the experiment for two aspects:

(1) The robustness of RDTrack in PIU recognition.
(2) The effectiveness of RDTrack with different parameters.

### 6.1. Implementation

#### 6.1.1. Hardware

We adopt an ImpinjR420 reader supporting four 9-dbi-gain directional antennas (9028PCL12NF). We use the Impinj H47 passive tags in our experiments. The frequency of the reader is 924.375 MHz, and the bandwidth is 231.25 kHz. Meanwhile, we set the reader mode as “DenseReaderM8” in order to have an evident Doppler shift and the maximum Signal-to-Noise Ratio (SNR). Finally, we use a digital video camera to get the real condition.

#### 6.1.2. Default Deployment Set

**Figure 11:**
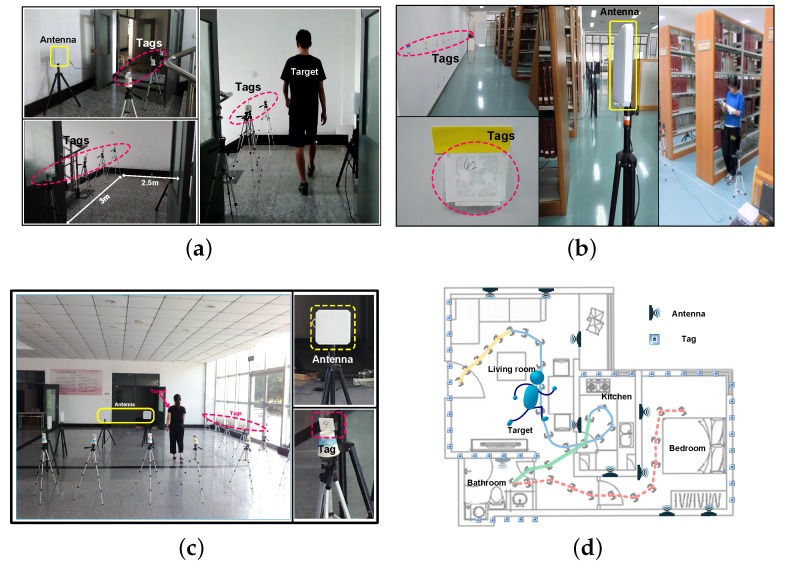
Different experimental environments. (**a**) RDTrack in the corridor; (**b**) RDTrack in the library; (**c**) RDTrack in the laboratory; (**d**) RDTrack in the apartment.

As shown in Figure 11c, we implement our experiment in an indoor environment with a size of about 5 m × 7 m. Two antennas arrays are deployed at two adjacent edges of the monitoring area, and tag arrays (with six tags) are deployed at two opposite sides of the area. The distance between each antenna and the tag arrays is 2.5 m. Each tag is at a distance of 2.5 m from other adjacent tags. There also are concrete walls, other obstacles and a PC, which is used for receiving the messages sent by the Impinj.

#### 6.1.3. Reference Profiles Setup

We build profiles for three different motions (turning, going straight, going backward) in walking. As is shown in Figure 11c, the distance between a set of the 9-dbi-gain antenna and five Impinj H47 passive tags is 2.6 m, and the target is walking among the tags and the antenna. Then, we can apply the profiles to recognize the motion in different environments. For each motion, we gather the profiles from a volunteer, whose height is 1.75 m with a medium body build. All the profiles are saved in the database and cost about 1.5 min. The specific details of each motion are shown in Table 1.

### 6.2. Effectiveness Validation

In this part, we are going to verify the effectiveness of RDTrack in different environments. In the same default deployment, experimental verification is performed in four different environments, including:

1. A small short corridor between staircases, which has a length and width of 2 m × 3.5 m, as shown in Figure 11a.
2. The reading room of the library on our campus, which has a length and width of 1 0 m × 12 m, as shown in Figure 11b.
3. The first floor of our laboratory building, which has a length and width of 5 m × 7 m, as is shown in Figure 11c.
4. A typical apartment consisting of a living room connected to the kitchen, bathroom and bedroom, which covers 8 m × 9 m, as shown in Figure 11d.

For every scene, we program a variety of paths in advance such as only straight, only turn 90${}^{\circ }$, only turn 180${}^{\circ }$ and uncertain path. Then, we ask the volunteer to walk through the path, and we obtained more than 1000 primitive data profiles in total. During the walking, the volunteer performs the motions in Table 1. At each path, the volunteer performs each motion five times. Meanwhile, we use the camera to collect the image data to verify the accuracy and practicability of our method.

#### 6.2.1. Segmentation Accuracy

Evaluation index: We use κ as the accuracy of the segmentation. The evaluation index is set up as follows:

(23) $$\kappa =\frac{overlap(P_{est},P_{act})}{P_{act}}$$

where $P_{est}$ is the estimated duration and $P_{act}$ is the actual duration of a motion. We use the overlap to calculate the degree of matching coverage of the two durations. The κ is proportional to the segmentation accuracy.

**Figure 12:**
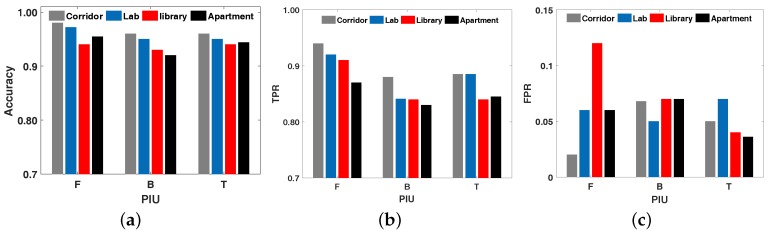
Under different environments. (**a**) Segmentation accuracy under different environments; (**b**) True positive rate under different environments; (**c**) False positive rate under different environments.

Result analysis: From the result we get from the experiment, the segmentation accuracy of RDTrack is between 96.9% and 100%, as is shown in Figure 12a. We can find that the accuracy of RDTrack in the laboratory is higher than others, because the laboratory environment is relatively simple; uncontrollable situations such as NLOS and through the wall are less than other environments. Therefore, we can obtain an accurate start time and end time after analysis. Furthermore, we can easily notice that the segmentation accuracy of “turning” is lower than “forwards” and “backwards”. The reason for this is that the motion is too similar to identify. The average accuracy of each motion is 98.0%, 97.42% and 97.0%. In summary, all motions have high segmentation accuracy. This can fully verify the robustness of the motion segmentation. In addition, we compare RDTrack with Rollcaller [11] and Twins [12] to see the accuracy of the affected time of the tag, as shown in Table 2. In our experiments, the distance between the antenna and the tag is 3 m and the speed of target movement is 0.9 m/s. From the view of Table 2, the tag discovery accuracy of RDTrack has a great performance, better than Rollcaller and Twins. Moreover, in real time, the delay time of RDTrack is less than 0.3 s, which includes the time of gathering two packets (≤20 ms) and the time of system computation (≤43 ms).

#### 6.2.2. Motion Recognition Accuracy

Evaluation index: We use two indexes to evaluate the motion recognition as follows:

(a) True Positive Rate (TPR): The accuracy of RDTrack. The proportion of the number of PIU that have been correctly identified to the total number of PIU.
(b) False Positive Rate (FPR): The proportion of the number of other activities that were misjudged as Activity B to the total number of Activity B.

**Figure 13:**
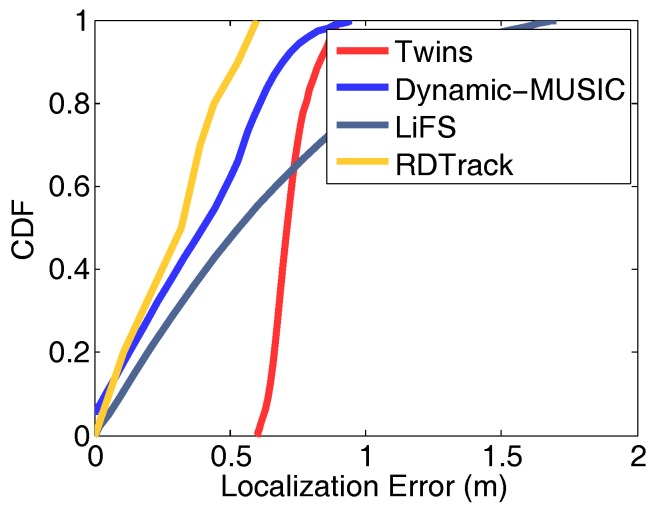
CDF plot of the localization errors.

Result analysis: In order to illustrate the effectiveness of our method more clearly, we calculate the specific values of TPR and FPR for different activities in different environments, as is shown in Figure 12b and Figure 12c. The TPR of the three motions in different environments is between 82.6% and 94.29%, and the average is 91.82%, 84.17% and 86.38%. The FPR of the motions is less than 13%, and the average is 6.51%, 6.45% and 4.91%. These analytical data show that the path matching accuracy of RDTrack is trustworthy. Our method is robust to different environments without learning before. Furthermore, we compare RDTrack with Twins [12], Dynamic-MUSIC [29] and LiFS [10], to see the tracking accuracy of RDTrack, as shown in Figure 13. Obviously, both TPR and FPR values fluctuate very little and remain essentially constant. So we confirm the suitability and accuracy of our experiments.

#### 6.2.3. System Performance

**Figure 14:**
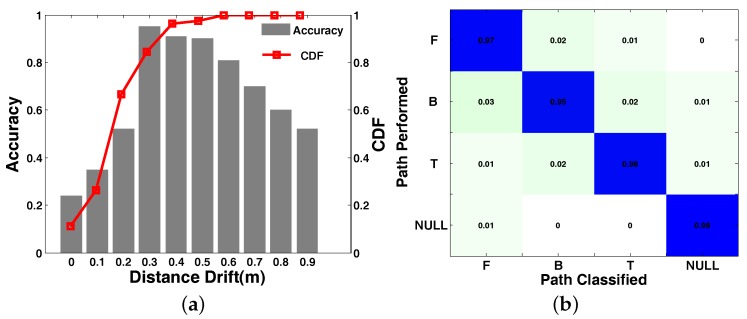
System performance. (**a**) Communication distance between antenna and tags; (**b**) Confusion matrix of motion recognition.

As shown in Figure 14a, we can figure out that 80% of anchor tags are measured within a 30-cm distance drift. This is an acceptable error when applying the anchor tag in real applications. Besides, the bars of accuracy in Figure 14a illustrate that most of the distance drift is concentrated in the interval of 30 cm–50 cm. This means that the extreme error occurs rarely. What is more, we analyze the tracking accuracy of RFTrack and compare it with RollCaller [11], Twins [12], Dynamic-MUSIC [29] and LIFS [10]. The comparative experiment was carried out in a general laboratory. There were desks, stools, computers and other daily necessities in the room. The indoor area was 8 m × 6.5 m. Under the same environment and deployment, we compared Twins, Dynamic-MUSIC, LiFS and our method. The results are shown in Figure 13. The average position error of our method is 0.33 m, while the error distribution of the other three methods is 0.71 m, 0.4 m and 0.52 m.

### 6.3. Evaluation under Different Parameters

#### 6.3.1. Impact of Human Diversity

**Figure 15:**
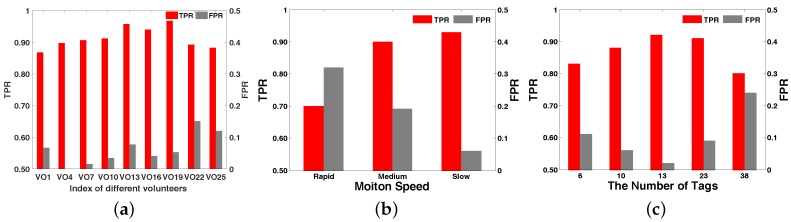
The comparison of TRP and FPR. (**a**) TPR and FPR under different volunteers; (**b**) TPR and FPR under different speeds; (**c**) TPR and FPR under different numbers of tags.

We used the default parameters, and 25 different volunteers participated in the experiment. These volunteers include 15 males and 10 females with ages in the range of 13–60. Their height distribution was 130 cm–185 cm, and their weight was 40kg–86 kg. There were middle school students, college students and retired people among them. We calculated the TPR and FPR, shown in Figure 15a. The accuracy rate was between 85.75% and 92.67%. Most of them had high accuracy of about 90%, and the average accuracy was about 89.67%. Moreover, VO13 (Volunteer No.13) and VO19 were higher than the others that were above 95%. This is because the body shape of the VO13 and VO19 volunteers was similar to the volunteers in the fingerprint library. Therefore, it was an effective strategy to increase the RDTrack fingerprint database to improve the accuracy of RDTrack path matching. Overall, the performance accuracy of RDTrack was above 85%. This shows that the different targets had less influence on the accuracy of motion recognition.

#### 6.3.2. Impact of the Motion Speed

We measured three different speeds: rapid (3 m/s), medium (1 m/s∼2.5 m/s) and slow (0.5 m/s), and the volunteers performed three basic movements at the different speeds 10 times. Then, we obtained the final results for the TPR and FPR, as are shown in Figure 15b. From the bar chart, we know that when the speed was below 2.5 m/s, the TPRs were more than 90% and the FPRs less than 20%. For the slow speed, the FPR was less than 9%. When accelerated, the TPR decreased and the FPR increased. This tells us that when the speed was smooth and steady, the accuracy of motion recognition was stable. Therefore, the RDTrack can be used for recognizing motions in most conditions.

#### 6.3.3. Impact of the Numbers of Tags

We measured a series of different numbers of label experiments under the default deployment. We let the volunteer (height 180 cm and weight 75 kg) walk through the experiment with different motions. The numbers of tags included 6, 10, 13, 23 and 38, in order to obtain the different numbers of labels’ experimental results. The distance between two adjacent labels was 0.25 m, 0.3 m, 0.5 m, 0.7 m and 1 m. The experimental results are shown in Figure 15c. Obviously, the TPR increased when the number of the tags increased, but decreased when the number of the tags went beyond 13. The FPR and the TPR were opposite. This shows that in a certain range, when we used more tags, the experiment results were more accurate. However, over this range, the accuracy would decrease rapidly. Finally, RDTrack worked efficiently when the tag number was approximately 13.

#### 6.3.4. Impact of Moving Direction

To examine the impact of moving direction on our method, we experimented with three moving direction (forward, backward, turning) in the default deployment. As is shown in Figure 14b, we established the confusion matrix for all the motions in three different situations. The row indicates the actual motion, and the column indicates the recognized motion. The confusion matrix shows that all three motion’s recognition accuracy was 97%, 95%, 92% and 94% and the average recognition accuracy for all the motions was 94.5%. The forward and backward motions were so similar that the probability of being mistaken for each other was larger than other movements.

#### 6.3.5. Impact of the Experimental Environment

We measured four different experimental environments, including a short corridor, a reading room, a laboratory and a lobby in the same deployment. As is shown in Figure 12a, the accuracy of these four environments was 89.5%, 92.5%, 91.75% and 91.3%. The accuracy was highest in the laboratory, while the accuracy in the corridor was lowest. This situation is due to two reasons: Firstly, in the corridor, the number of barriers was more than other environments and the effective detection area was less than the others. Secondly, when people walked through the detection area, their bodies blocked the information exchange between the tags and antenna, so the reader could not receive the tag’s signals.

### 6.4. Supplemental Experiment

#### 6.4.1. Impact of the Number of Targets

To examine the impact of the number of targets on RDTrack’s performance, we let two volunteers walk on different paths through the detection area. The result showed us that when the distance between two volunteers was beyond 1 m the recognized accuracy of both volunteers was about 90%. However, if the distance of two volunteers were too short, the recognized accuracy would rapidly decrease, for one of them will be affected by the others.

#### 6.4.2. Impact of Multiple Readers

We used more than one reader in out default deployment setting to detect the average accuracy over different motions. When the number of reader was only one, the accuracy of RDTrack was 91%. Increasing the number of reader to two, the accuracy of RDTrack was 97%, and reached 99% when there were three readers. However, the accuracy of RDTrack would not increase when there were four or more readers.

## 7. Related Work

Target tracking systems generally adopt all kinds of techniques such as computer vision [2], infrared electromagnetic radiation [4], ultra-sound [3] and inertial sensors [30]. However, these techniques suffer from many limitations such as sensitivity to lighting, requiring line-of-sight communication between the target and the device, high installation and demanding extra sensors or devices to be worn or installed. These limitations promoted the exploitation of RF-based device-free target tracking systems, which were based on analyzing the changes in the characteristics of the wireless signals, such as RSSI, CSI and Doppler, caused by human motion.

(1) RSSI and CSI-based systems: Device-free location and PIU recognition relying on RSSI fluctuations or the more detailed Channel State Information (CSI) in WiFi networks have emerged as a ubiquitous solution for presence detection [31,32] and tracking [7,8,9]. For tracking systems, ACE [9] introduces a system that uses a probabilistic energy-minimization framework that combines a conditional random field with a Markov model to capture the temporal and spatial relations between the entities’ poses. In [8], the authors proposed 3D ray tracing as a way to generate a highly accurate radio map automatically. In addition, Pilot [7] designed an essential anomaly detection block as a localization trigger relying on the CSI feature shift when an entity emerges. The work in [33] introduced an algorithm, BFVP, which provides a reliable localization precision. This algorithm is Bayesian filter-based, which can be used in different complex environments. The work in [34] showed us a fine-scale motion capture and localization system, which combines RF-based and non-RF-based observation together to achieve a high accuracy range. The work in [35] explored three different filtering algorithms that enhance the tracking performance and form a stable location system.

These solutions described above typically require much prior training and mass deployment. If not, one cannot guarantee precision because of multipath and non-line-of-sight paths [36].

(2) Doppler-based systems: Some research works have also investigated the possibility of using Doppler to realize tracking and inspection activities. WiSee [37] leverages the Doppler shifts of WiFi signals reflected by human body motions to distinguish nine different gestures. In addition, RollCaller [11] does indoor navigation with the key insight that a person’smovement has an impact on the Doppler values collected from the objects when getting close to the tag. What is more, Twins [12] leverages a newly-observed phenomenon caused by interference among passive tags, presents a new interference model to explain it and uses extensive deployment to validate it.

While promising, these techniques suffer from many limitations such as requiring mass deployment and ceaseless calibration [12]. Specifically, none of these systems can provide the fine-grained movement locus of the target. RDTrack builds on this foundational work to achieve a fine-grained tracking system based on clean Doppler changes calculated from the clean phase changes, without any training, mass deployment nor ceaseless calibration.

## 8. Conclusions

In this paper, we present an accurate device-free tracking system using inexpensive commercial off-the-shelf RFIDs, called RFTrack. Without requiring any training prior to deployment and not requiring any modification to the available equipment, RDTrack addresses system challenges including using the DWT technique to detect the beginning and the end affected time of tags, which are caused by the target motion, and based on the HMM model, we presented a less complex trajectory estimation model. This model can achieve a high tracking accuracy. RDTrack consists of three steps, which include signal revision, signal denoising and trajectory smoothing. Our results show that in four different scenes, RDTrack detects the basic pattern with an average accuracy of 91% using only a single RDTrack. Currently, we are extending the system to standard WiFi, which is facing a challenge in extracting clean phase values. 

## Figures and Tables

**Table 1:** The motion information of RDTrack.

Abbreviations	Motion	Training Time
F	Going Straight	10.23 s
B	Going Backward	10.43 s
T	Turning	2.47 s

**Table 2:** The accuracy of the affected time of the tag.

System	Time Delay	Tag Discovery Accuracy
RDTrack	≤0.3 s	100%
Rollcaller	≤0.5 s	95%
Twins	≤1.1 s	98.8%

## Abbreviations

The following abbreviations are used in this manuscript:

RFID	Radio Frequency Identification
RF	Radio Frequency
RSSI	Received Signal Strength Indicator
CSI	Channel State Information
FMCW	Frequency-Modulated Continuous Wave
USRP	Universal Software Radio Peripheral
DWT	Discrete Wavelet Transform
PIU	Path Information Unit
UHF	Ultra-High Frequency
RSS	Received Signal Strength
V	Velocity
HMM	Hidden Markov Model
IoT	Internet of Things
